# Draft Genome Sequence of Deep-Sea *Alteromonas* sp. Strain V450 Isolated from the Marine Sponge *Leiodermatium* sp.

**DOI:** 10.1128/genomeA.01508-16

**Published:** 2017-02-02

**Authors:** Guojun Wang, Nolan H. Barrett, Peter J. McCarthy

**Affiliations:** Harbor Branch Oceanographic Institute, Florida Atlantic University, Fort Pierce, Florida, USA

## Abstract

The proteobacterium *Alteromonas* sp. strain V450 was isolated from the Atlantic deep-sea sponge *Leiodermatium* sp. Here, we report the draft genome sequence of this strain, with a genome size of approx. 4.39 Mb and a G+C content of 44.01%. The results will aid deep-sea microbial ecology, evolution, and sponge-microbe association studies.

## GENOME ANNOUNCEMENT

*Alteromonas* (*Gammaproteobacteria*, *Alteromonadales*, *Alteromonadaceae*), a genus of Gram-negative *Gammaproteobacteria*, is often found in seawater, and is attracting research interest in the areas of ecology, evolution, and secondary metabolism (1–4). So far, the genome sequences of 42 *Alteromonas* species/strains have been deposited into public databases, and 18 of these were released in 2016 (5).

Here, we report the draft genome sequence of a deep-sea *Alteromonas* sp. strain V450. This strain was cultured using mucin-amended seawater from the Lithistid sponge *Leiodermatium* sp., the producer of the potent antimitotic agent leiodermatolide (6, 7). The sponge was collected at a depth of 392.5 m on the Miami Terrace located in the southeast of Miami, FL (8).

The V450 genome is the first reported genome of an *Alteromonas* strain derived from a marine sponge. The 16S rRNA gene sequences of V450 shared 99% similarity with those of various *Alteromonas macleodii* strains, including the deep-sea isolates AltDE1 and UM7 (3, 9).

Genome sequences were obtained using the Illumina HiSeq 2500 sequencing platform; the draft genome was obtained from 15 million reads of paired-end 125 (PE125) via assembling and optimization according to paired-end and overlap relationships. The G+C content was calculated by GC-depth analysis. The reads were assembled *de novo* by ABySS (10), SPAdes (11), and MIX (12). Based on the assembly results, the number of scaffolds is five, and the total coverage over the genome is 1,225-fold. The *N*_50_ value is 4,393,372 bp. The majority of the sequences were assembled into a scaffold of over 4.3 Mb; with additional four small contigs, the total genome of V450 is 4,398,017 bp. The G+C content is 44.01%. The protein-coding open reading frames (ORFs) and functional annotation were predicted by RAST and Prokka (13, 14); rRNA was predicted by the rRNA database and RNAmmer (15), and tRNA was predicted by tRNAscan (16). A total of 3,795 ORFs, 52 tRNAs, and six rRNAs are predicted.

In order to determine whether V450 is possibly the microbial producer of leiodermatolide associated with *Leiodermatium* sponge, antiSMASH (17) was run to predict secondary metabolic gene clusters in the V450 genome. A total of 25 predictive gene clusters are present in the V450 genome for the biosynthesis of secondary metabolites, such as bacteriocin and nonribosomal peptide.

### Accession number(s).

This whole-genome shotgun project has been deposited at DDBJ/ENA/GenBank under the accession no. MODU00000000. The version described in this paper is version MODU01000000.
